# SARS-CoV-2 BNT162b2 vaccine–induced humoral response and reactogenicity in individuals with prior COVID-19 disease

**DOI:** 10.1172/jci.insight.155889

**Published:** 2022-02-22

**Authors:** Steven G. Kelsen, Alan S. Braverman, Mark O. Aksoy, Jacob A. Hayman, Puja S. Patel, Charu Rajput, Huaqing Zhao, Susan G. Fisher, Michael R. Ruggieri, Nina T. Gentile

**Affiliations:** 1 Department of Thoracic Medicine and Surgery,; 2 Department of Anatomy,; 3 Department of Biomedical Education and Data Science, and; 4 Department of Emergency Medicine, Lewis Katz School of Medicine at Temple University, Philadelphia, Pennsylvania, USA.

**Keywords:** COVID-19, Adaptive immunity

## Abstract

**BACKGROUND:**

Most individuals with prior COVID-19 disease manifest long-term protective immune responses against reinfection. Accordingly, we tested the hypothesis that humoral immune and reactogenicity responses to a SARS-CoV-2 mRNA vaccine differ in individuals with and without prior COVID-19 disease.

**METHODS:**

Health care workers (*n* = 61) with (*n* = 30) and without (*n* = 31) prior COVID-19 disease received two 30 μg doses of Pfizer BNT162b2 vaccine 3 weeks apart. Serum IgG antibody against the spike receptor-binding domain; serum neutralizing activity; and vaccine reactogenicity were assessed longitudinally every 2 weeks for 56 days after the first injection.

**RESULTS:**

The COVID-19 group manifested more rapid increases in spike IgG antibody and serum neutralizing activity after the first vaccine dose but showed little or no increase after the second dose compared with the infection-naive group. In fact, spike IgG was at its maximum level after the first dose in 36% of the COVID-19 group versus 0% of the infection-naive group. Peak IgG antibody levels were lower but appeared to fall more slowly in the COVID-19 group versus the infection-naive group. Finally, adverse systemic reactions, e.g., fever, headache, and malaise, were more frequent and lasted longer after both the first and second injection in the COVID-19 group than in the infection-naive group.

**CONCLUSION:**

Individuals with prior COVID-19 disease demonstrate a robust, accelerated humoral immune response to the first dose but an attenuated response to the second dose of BNT162b2 vaccine compared with controls. The COVID-19 group also experienced greater reactogenicity. Humoral responses and reactogenicity to BNT162b2 differ qualitatively and quantitatively in individuals with prior COVID-19 disease compared with infection-naive individuals.

**FUNDING:**

This work was supported by Temple University institutional funds.

## Graphical abstract

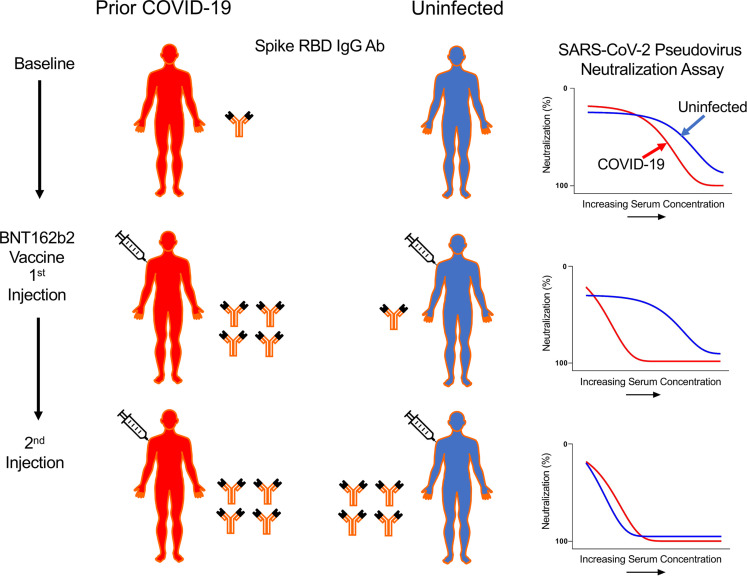

## Introduction

Vaccination against the SARS-CoV-2 virus provides a way to rapidly achieve widespread protective immunity of the uninfected population and, thus, end the COVID-19 pandemic. In fact, phase II/III clinical trials of the now in use Pfizer BNT162b2 and Moderna mRNA-1273 mRNA vaccines demonstrate 90%–95% protection against SARS-CoV-2 infection and near 100% effectiveness in preventing severe COVID-19 disease, e.g., hospitalization or death (1–5). Similar protective efficacy of the Pfizer BNT162b2 vaccine has been reported in a country-wide population study in Israel (3).

Public health authorities in the US recommend full vaccination, including both doses of the 2-dose regimen mRNA vaccines, for all those over 12 years of age, including those with prior SARS-CoV-2 infection or COVID-19 disease (6). Interestingly, this recommendation has been made despite the fact that trials of both COVID-19 mRNA vaccines excluded volunteers with a history of COVID-19 disease, and most individuals have durable immune memory after COVID-19 (7–12). Moreover, the rate of SARS-CoV-2 reinfection in the 15 months since the pandemic started has been quite low (13–15).

Of interest in this regard, several studies have demonstrated that individuals with prior SARS-CoV-2 infection/COVID-19 disease exhibit rapid and robust humoral and cell-mediated immune responses to a single dose of a 2-dose mRNA vaccine regimen (16–28). Moreover, responses of those with prior SARS-CoV-2 infection to the first dose greatly exceed the responses of infection-naive individuals. This observation has led to the suggestion that a single dose of a COVID-19 mRNA vaccine may be sufficient to provide adequate protection against infection for individuals with prior SARS-CoV-2 infection (19, 25). Given the scarcity of vaccines in most of the world, it has been suggested that individuals with a history of prior SARS-CoV-2 infection receive only 1 dose of the vaccine (19, 21, 23, 25). Using this approach, the second dose would be withheld until some future date as needed.

In contrast to the considerable available data defining the response to the first dose of an mRNA vaccine in individuals with prior SARS-CoV-2 infection/COVID-19 disease, little or no information is available regarding their response to the second dose of vaccine given at the standard 3- to 4-week dosing interval.

Accordingly, this longitudinal study examined the hypothesis that the time course and magnitude of the humoral immune response and reactogenicity induced by a full 2-dose mRNA vaccination regimen differed quantitatively in individuals with prior COVID-19 disease versus infection-naive individuals. Specifically, we examined the level of anti-spike receptor-binding domain (RBD) IgG antibody and serum neutralizing activity serially at fixed points in time, i.e., 2-week intervals, for 56 days after the first and 35 days after the second injection in SARS-CoV-2–infected and infection-naive health care workers from the same academic health care center. To avoid possible confounding effects of differences in immune potency and reactogenicity between mRNA vaccines, responses to a single vaccine i.e., Pfizer BNT162b2, were studied (29).

Our results indicate that spike RBD IgG antibody levels and serum neutralizing activity in response to BNT162b2 vaccine were time dependent and increased more rapidly while reaching lower peak levels and appeared to fall more slowly in individuals with prior COVID-19 disease than in infection-naive individuals. Our data also represent the first available set to our knowledge of time-dependent normal values for spike RBD IgG antibody induced by BNT162b2 in normal adults with and without prior COVID-19 disease.

## Results

### Study population.

Demographic characteristics of individuals with prior COVID-19 infection (COVID-19 group) (*n* = 30) and the control group (*n* = 31) are shown in Table 1. The 2 groups were well matched for age, sex, and ethnicity. Mean age in the COVID and control groups was 47 years and 45 years, respectively (*P* > 0.80). Sex was male in 50% of the COVID-19 group and 52% of the control group (*P* > 0.50). Most individuals were White (80% and 77%, in the COVID-19 and control groups, respectively, *P* > 0.90), but African-American and Asian individuals were also included in both groups.

Clinical features of the COVID-19 illness are shown in Table 2. Most individuals (93%) were symptomatic; 7% were asymptomatic. Four individuals (13%) had COVID-19 pneumonia; 2 (7%) were hospitalized.

On average, individuals in the COVID-19 group received the first dose of vaccine approximately 7 months after onset of symptoms (POS) (mean ± SEM, 201 ± 16 days; range, 25–332 days) (Table 2). In fact, 58% of the COVID-19 group received the first vaccine dose more than 7 months POS (i.e., > 220 days).

Prevaccination levels of spike protein RBD IgG or nucleocapsid IgG antibodies were elevated in 81% of individuals in the COVID-19 group and none of the controls (Supplemental Figure 1; supplemental material available online with this article; https://doi.org/10.1172/jci.insight.155889DS1).

### Antibody responses to vaccine.

**Figure 1:**
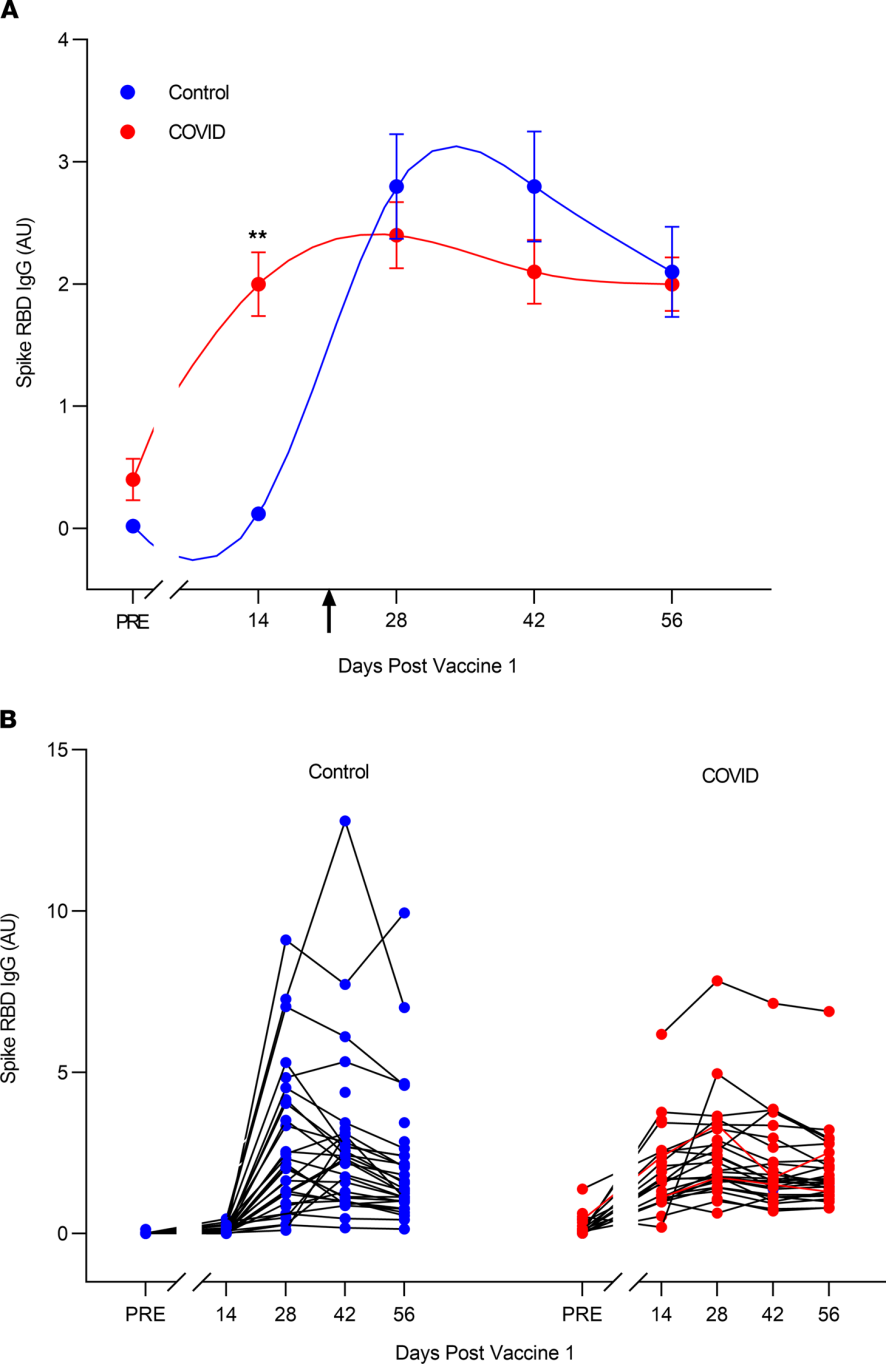
Spike RBD IgG antibody responses to the BNT162b2 vaccine in COVID-19 and control groups. (**A**) Group mean ± 1 SEM responses. Vertical arrow indicates time of second vaccine injection. Note that the time course of spike RBD IgG antibody response to vaccination was significantly different between COVID-19 and control groups (*P* < 0.0001; linear mixed-effects model for repeated measures). Differences in spike IgG antibody levels were significant prevaccine (*P* < 0.0003) and at day 14 (**P < 0.0002). However, spike IgG levels were similar at days 28, 42, and 56 (*P* > 0.10 for all 3 comparisons). Sample size in the COVID-19 group was as follows: day 14 (*n* = 25), day 28 (*n* = 28), day 42 (*n* = 28); and day 56 (*n* = 28). Sample size in the control group was as follows: day 14 (*n* = 25), day 28 (*n* = 28), day 42 (*n* = 30), and day 56 (*n* = 31). (**B**) Spike RBD IgG antibody responses to vaccine in individuals with prior COVID-19 disease and control individuals. Note the considerable interindividual variability in both groups.

Spike RBD IgG antibody levels at each time point after vaccination are shown as arbitrary units in Figure 1 and as μg protein/mL in Table 3.

The time course and magnitude of the spike RBD antibody response differed greatly across individuals in both groups (Figure 1B) but were significantly different in the COVID-19 and control groups as a whole (*P* < 0.0001 by linear mixed effects; Figure 1A). In the COVID-19 group, spike RBD IgG increased more rapidly, peaked earlier, and appeared to fall more slowly than in the control group.

Specifically, in the COVID-19 group, spike RBD IgG antibody at day 14 after first injection increased significantly from the prevaccine level (*P* < 0.0001) (Table 3). Thereafter, little or no increase in spike IgG occurred in the COVID-19 group, and IgG values at subsequent time points were not statistically significantly different from the day 14 value (*P* > 0.05 for all later time points compared with day 14). In the COVID-19 group, spike RBD IgG levels peaked at day 14 in 36% of individuals; at day 28 in 52% of individuals; at day 42 in 12% of individuals; and at day 56 in 0% of individuals (Figure 1B).

In contrast, in the control group, increases in spike RBD IgG at day 14 were not statistically different from the prevaccine value (*P* > 0.20), and levels were significantly less than in the COVID-19 group (*P* < 0.0002). Thereafter, spike RBD IgG levels increased markedly between day 14 and day 28 in the control group (*P* < 0.0001 for comparison of the 2 time points). In the control group, spike RBD IgG peaked at day 14 in 0% of individuals; at day 28 in 60% of individuals; at day 42 in 37% of individuals; and at day 56 in 3% of individuals. Of note, peak spike RBD IgG values were significantly greater in the control than the COVID-19 group (*P* < 0.0002) (Table 3).

Spike RBD IgG antibody levels declined from peak values in both groups but appeared to do so more slowly in the COVID-19 group. In particular, from day 42 to day 56, spike RBD IgG fell significantly in the control group (*P* < 0.001) but not in the COVID-19 group (*P* > 0.90).

**Figure 2:**
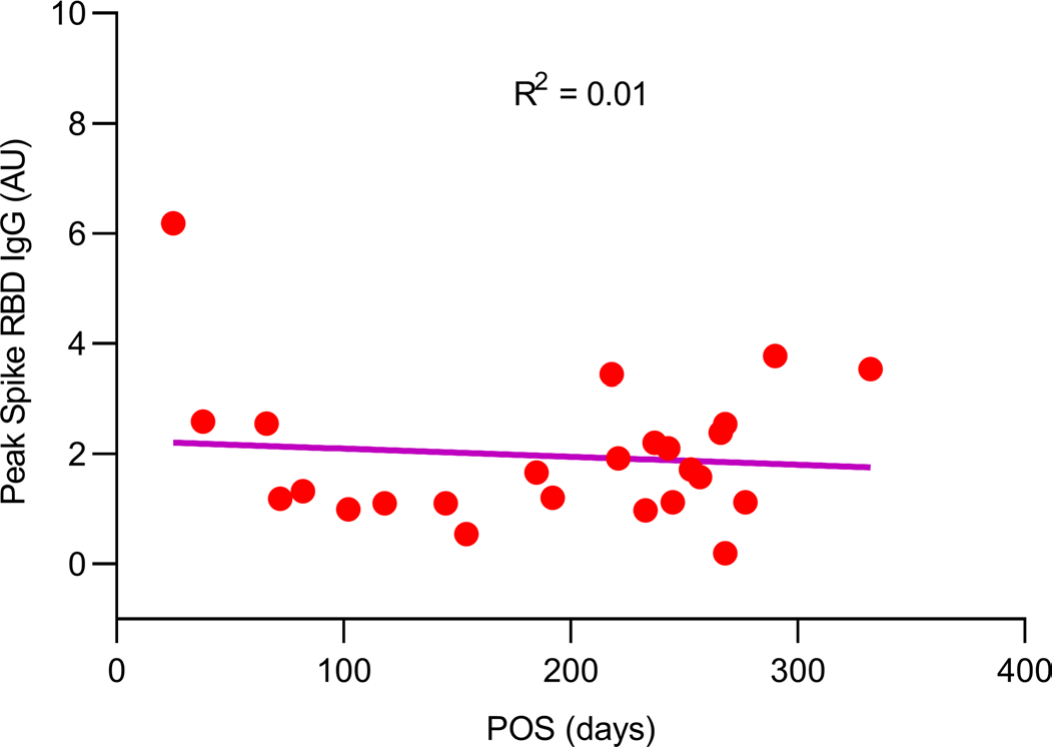
Relationship between vaccine-induced peak spike RBD IgG antibody and interval after onset SARS-CoV-2 symptoms in individuals with COVID-19. There was no discernible relationship (*r*^2^ = 0.01 by linear regression). Of note, 2 of the 29 individuals in the COVID-19 group were asymptomatic. Hence, no after onset of SARS-CoV-2 symptoms (POS) value is available.

The considerable variation in the interval between SARS-CoV-2 illness and vaccination i.e., POS, in the COVID-19 group, i.e., 25–332 days (Table 2) did not appear to explain differences in peak spike RBD IgG levels. In fact, the peak spike RBD IgG level was unrelated to the interval between POS and vaccination (Figure 2; *r*^2^ = 0.01 by linear regression).

In contrast to spike RBD IgG, nucleocapsid IgG antibody, which was assessed to detect the unlikely possibility of superimposed SARS-CoV-2 infection during the study, was undetectable in the control group and declined progressively in the COVID-19 group (Supplemental Figure 2A). Individual nucleocapsid IgG values are also shown in Supplemental Figure 2B.

### Serum neutralization activity.

**Figure 3:**
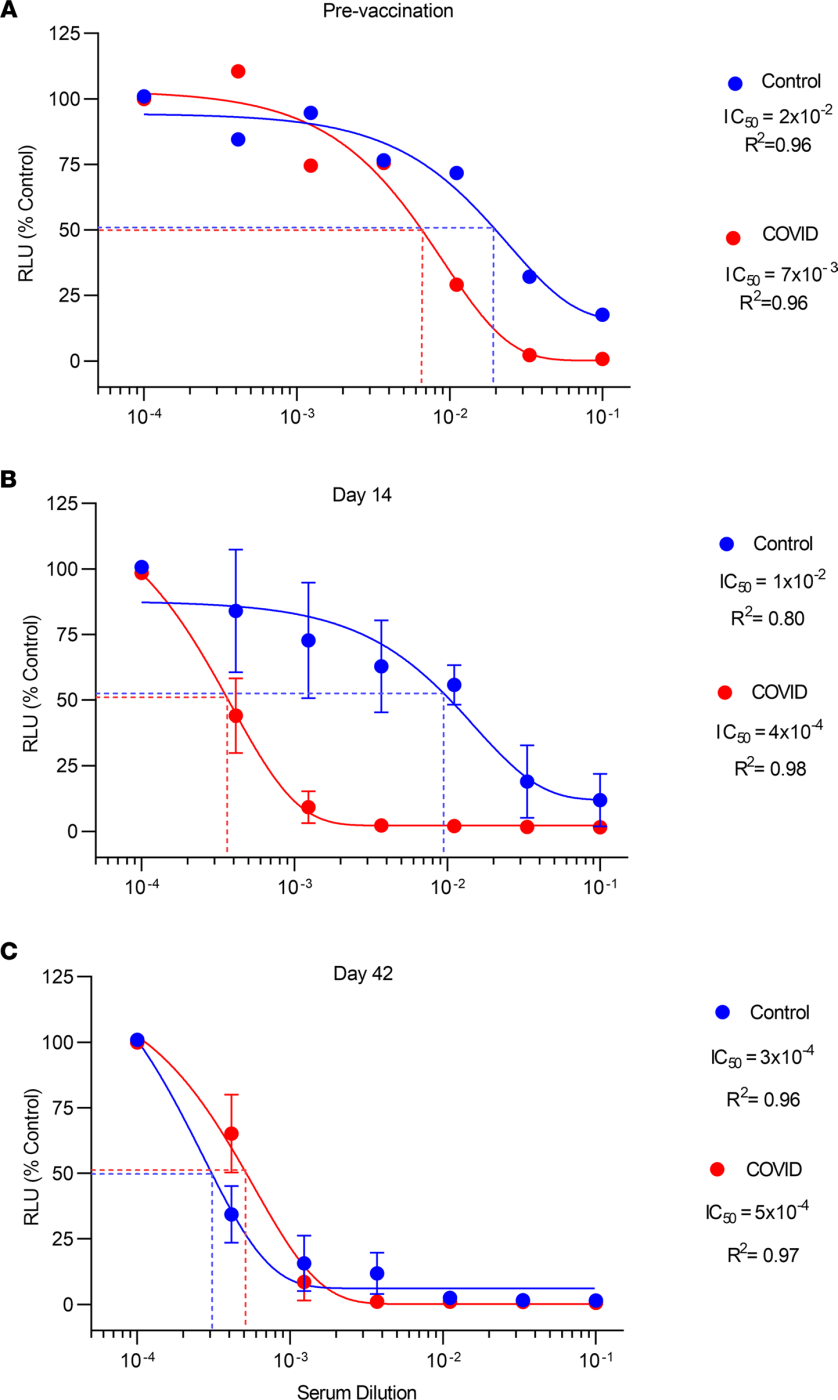
Serum neutralizing activity in COVID-19 and control groups. Pseudovirus uptake in HEK293 ACE2-overexpressing cells was assessed from luciferase activity, i.e., relative light units (RLU), and is shown on the *y* axis. Serum dilution is shown on the *x* axis. The 100% control value on the *y* axis represents maximal virus uptake occurring in the absence of serum. IC_50_ was calculated using sigmoidal 4-factor polynomial, nonlinear regression. Data are shown prevaccination (**A**) and day 14 (**B**) and day 42 (**C**) after first vaccine dose. Prevaccine, neutralizing activity in the COVID-19 group (*n* = 7) tended to be greater than that in the control group (*n* = 7) (IC_50_ 7 × 10^–3^ vs. 2 × 10^–2^ dilution, respectively) but was not statistically significantly different (*P* = 0.20 by 2-way ANOVA). At day 14 after first injection, neutralizing activity increased greatly in the COVID-19 group (*n* = 21) but was unchanged in the control group (*n* = 21) (IC_50_ = 3.6 × 10^–4^ vs. 1 × 10^–2^ dilution, respectively; *P* < 0.03 by 2-way ANOVA for comparison of the 2 groups). In contrast, at day 42, neutralizing activity increased greatly in the control group (*n* = 21) but only slightly in the COVID-19 group (*n* = 21) (IC_50_ = 5 × 10^–4^ vs. 3 × 10^–4^ dilution, respectively) and was again not statistically significantly different (*P* = 0.11 for comparison of the 2 groups by 2-way ANOVA). For days 14 and 42, the same 21 individuals with prior COVID-19 disease and 21 control individuals were studied, and for both groups each data point is the mean ± SEM for 3 pools of 7 individuals each.

Prevaccination, serum neutralization activity was modestly elevated in the COVID-19 versus control group (50% inhibition [IC_50_], 7 × 10^–3^ dilution and 2 × 10^–2^ dilution, respectively) but was not statistically significantly different in the 2 groups (*P* = 0.20 by 2-way ANOVA) (Figure 3A).

At day 14 after the first injection, neutralizing activity increased approximately 17-fold in the COVID-19 group but was unchanged in the control group (Figure 3B). Specifically, IC_50_ was found at the level of 4 × 10^–4^ dilution in the COVID-19 group and at 1 × 10^–2^ dilution in controls, i.e., approximately 25-fold greater in the COVID-19 group (*P* < 0.03 by 2-way ANOVA).

However, at day 42 after first injection, neutralizing activity in the COVID-19 group was essentially unchanged from the 14-day value (i.e., IC_50_, 4 × 10^–4^ dilution and 5 × 10^–4^ dilution for days 14 and 42, respectively). In contrast, neutralizing activity increased markedly in the control group from the day 14 value (i.e., IC_50_, 1 × 10^–2^ dilution and 3 × 10^–4^ dilution for days 14 and 42, respectively) (Figure 3C). As a result, there were no differences in neutralizing activity in the COVID-19 and control groups at day 42 (*P* = 0.11 by 2-way ANOVA for the 2 curves).

### Vaccine reactogenicity.

**Figure 4:**
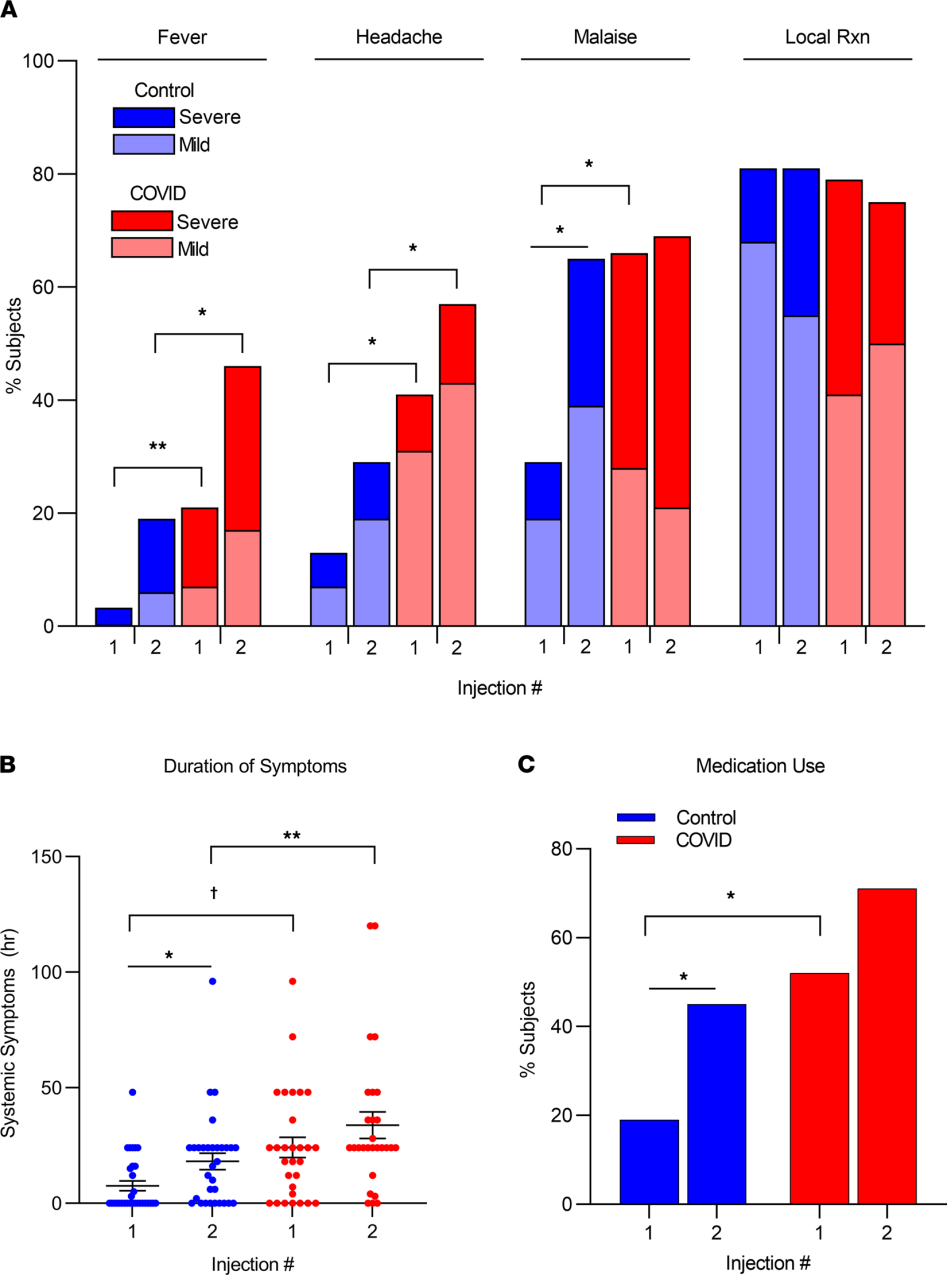
Reactions to the first and second Pfizer BNT162b2 mRNA vaccine in COVID-19 and control groups. (**A**) Prevalence and severity of systemic and local reactions. (**B**) Duration of systemic symptoms (mean ± 1 SEM for each group). (**C**) Frequency of medication use. Dark color (red or blue) indicates reaction scores of more than 6 severity; light colors indicate scores of equal to or less than 5. Brackets indicate statistical comparisons across groups assessed by Fisher’s exact test. Lines indicate comparisons within groups assessed by McNemar’s test for paired comparisons. *P < 0.05; **P < 0.01; **†***P* < 0.001. For all comparisons, the COVID-19 group, *n* = 30; control group, *n* = 31.

In general, systemic reactogenicity was greater in the COVID-19 group than the control group. Specifically, after the first injection, systemic symptoms (i.e., fever, headache, malaise/fatigue) were more frequent (*P* < 0.05 for each by Fisher’s exact test) and lasted longer (*P* < 0.001 by unpaired *t* test) in individuals in the COVID-19 group than in individuals in the control group (Figure 4, A and B). The use of antipyretics and/or analgesics was also more frequent in the COVID-19 group (*P* < 0.05 by Fisher’s exact test) (Figure 4C).

In contrast, local reactions (i.e., pain and tenderness) occurred in most individuals (≥80%) and with similar frequency in both groups (*P* = NS) (Figure 4A).

After the second injection, fever and headache were again more frequent in the COVID-19 group (*P* < 0.05 for both), and systemic symptoms continued to last longer (*P* < 0.01) (Figure 4, A and B). Local reactions to the second injection were similar in frequency and severity in both groups (Figure 4A).

All reactions resolved within 7 days without need for medical attention.

## Discussion

This study of a cohort of healthcare workers at a single academic medical center was designed to test the hypothesis that humoral immune responses and reactogenicity to a SARS-CoV-2 mRNA vaccine (BNT-162b2) differ in individuals with and without prior SARS-CoV-2 infection. This hypothesis was based on observations that individuals with prior SARS-CoV-2 infection have long-lasting memory B cell– and T cell–based immunity to the spike protein immunogen in the vaccine (7, 10). Accordingly, to define the vaccine-induced humoral immune response and reactogenicity, individuals were studied at 2-week intervals for 56 days after initial vaccination. The longitudinal design and length of the study allowed the onset, maximum response, and initial decay rate of spike RBD IgG antibody to be assessed in each individual.

Our results indicate that the time course and magnitude of the spike RBD IgG and serum neutralizing responses to the vaccine differed in the 2 groups. Specifically, in the COVID-19 group, spike RBD IgG antibody increased more rapidly but reached lower peak levels and seemed to fall more slowly, i.e., the response was “flatter” in the COVID-19 group than in the infection-naive control group. In fact, a large percentage of the COVID-19 group (36%) achieved maximum spike IgG antibody responses 14 days after the initial injection and did not respond to the second injection. Moreover, for the COVID-19 group as a whole, serum-neutralizing activity was at its maximum at day 14 and did not increase with the second vaccine. Accordingly, serum neutralizing activity mirrored the spike RBD IgG antibody response.

In contrast to changes in spike RBD IgG, nucleocapsid IgG was undetectable in the control group and decreased progressively over the observation period in the COVID-19 group. This finding rules out the remote possibility that superimposed SARS-CoV-2 infection in either group during the vaccination period may have confounded the results.

The frequency and duration of systemic reactions to the BNT-162b2 vaccine were heightened in individuals with prior SARS-CoV-2 infection although none were serious. Heightened systemic reactions in the COVID-19 group were present with both the first and second injection but were most apparent after the first injection.

The rapid, robust spike RBD IgG response to the first dose of vaccine in the COVID-19 group more than 7 months after prior infection and attenuated response to the second dose likely represent an anamnestic response mediated by long-duration memory B and T cells (7, 10, 12, 18). In fact, most SARS-CoV-2–infected individuals continue to have circulating memory B cells, bone marrow plasma cells, T follicular helper cells, and CD4 and CD8 Th1 cells present for 8 to 11 months after infection (7, 8, 10, 12). Preexisting immune memory against the spike immunogen in the COVID-19 group may also explain the heightened vaccine reactogenicity.

Our study has a number of strengths. First, the longitudinal study design with fixed 2-week sampling intervals and long duration allowed us to define the onset, peak, and initial decay rate of spike IgG antibody in both groups despite the considerable individual variability.

Second, accepted methods were utilized to assess spike RBD IgG antibody levels (30–35). In fact, antibody levels were expressed in absolute units (i.e., μg protein/mL) as well arbitrary units (i.e., chemiluminescence units) (32, 33). We specifically chose to express the spike IgG response as μg protein/mL to allow the “normal” vaccine response of healthy individuals to be available for medical decision making. That is, spike IgG levels generated in a healthy population at discrete points in time after vaccination are now available to assess the adequacy of the vaccine response in individuals with comorbid medical conditions that could impair the immune response (e.g., solid organ transplant) (36).

Third, the COVID-19 and control groups were well-matched for age and sex. This is important because age and sex determine immune responses to many vaccines (37, 38). Accordingly, differences in vaccine responses observed in the 2 groups in this study are not explainable by differences in age or sex.

We assessed the serum neutralizing activity in the 2 groups using an accepted pseudotyped lentivirus neutralization assay because not all antibodies targeting the RBD are neutralizing (39–41). Conversely, antibodies against spike protein epitopes outside the RBD may also be neutralizing (39, 40). Accordingly, serum neutralizing activity represents a more comprehensive way of assessing the humoral immune response (41).

Fourth, because both the immune response and adverse effects induced are vaccine-type dependent, a single, extensively used vaccine i.e., BNT162b2, was studied to avoid confounding effects of differing vaccines (29).

Our study also has limitations. First, while our data over 56 days after first injection suggest that spike IgG antibody levels may fall more slowly in the COVID-19 group than in infection-naive individuals, the long-term IgG antibody level was not defined in this study. This is of importance because vaccine protection depends on the sustained antibody level (38). Additional time points will be needed in this regard.

Second, we studied individuals in the COVID-19 group at a single time point i.e., approximately 7 months after infection. Although no relationship between the interval after infection and peak spike IgG antibody level was evident in the COVID-19 group, it would be desirable to obtain data at additional intervals after infection. In addition, our COVID-19 group consisted almost entirely of symptomatic individuals (93%) biased toward the severe end of the spectrum (several had pneumonia and were hospitalized). Because the intensity of the humoral and cell-mediated responses to SARS-CoV-2 correlate directly with COVID-19 symptoms, individuals with milder forms of SARS-CoV-2 infection, e.g., asymptomatic or pauci-symptomatic infection may respond differently (20, 42).

Third, our neutralizing assay utilized the original Wuhan-Hu-1 strain of the spike protein as the neutralizing antibody target. Mutations in the SARS-CoV-2 virus spike domain, however, make some virus variants, such as B.1.351 and B.1.1.7, more resistant to serum neutralization following natural infection or vaccination than the Wuhan strain (26–28, 43). Accordingly, our serum neutralizing data cannot be extrapolated to the recent SARS-CoV-2 variants of public health concern.

Finally, we did not assess vaccine-induced, IgG antibody–mediated effects on the innate immune system, such as opsonization, complement fixation, and NK cell activation. Of interest in this regard, Bartsch et al. observed in individuals convalescing from SARS-CoV-2 infection that above a threshold of approximately 0.5–1.0 μg/mL IgG spike RBD antibody protein, increasing antibody levels indicate broad activation of the adaptive and innate immune system (32). Specifically, Bartsch et al. observed that spike RBD IgG antibody levels above this threshold correlate directly with increasing neutrophil phagocytosis, complement fixation, and T cell responses to SARS-CoV-2 spike antigens. Of note, in our study, the spike RBD IgG antibody level in the COVID-19 group at day 14 after vaccination was approximately 45 μg/mL, suggesting that broad immune activation was achieved before the second injection.

The results of our study in individuals with prior infection are in agreement with recent studies of the immune response to the mRNA vaccines, most of which were cross-sectional in design and focused on the response to the first injection (16–28). As in present study, they also report more rapid and robust increases in spike RBD IgG antibody and serum neutralizing activity after the first injection of an mRNA vaccine in individuals with prior COVID-19 disease than in infection-naive individuals. In addition, serum neutralizing activity against variants of concern, such as B.1.1.7, B.1.351, and P.1, has been shown to increase significantly after the first dose of mRNA vaccine in individuals with prior SARS-CoV-2 infection (26–28). Our study extended these observations by more precisely defining the time course of the spike antibody response, i.e., onset, peak, and initial rate of decay to both doses of an mRNA vaccine. Moreover, our data in Table 3 represent to our knowledge the first available set of time-dependent “normal” values for spike IgG antibody induced by BNT162b2 in healthy individuals with and without prior COVID-19 disease. Accordingly, our data on minimal responses in the 2 groups can be used to assess the appropriateness of the anti-spike RBD antibody response to BNT162b2 in potentially immunocompromised individuals, e.g., solid organ transplant recipients (36). Our data may, therefore, may facilitate medical decision making with regard to the vaccine response in individual patients.

An important public health implication of our study is that individuals with a prior history of SARS-CoV-2 infection/COVID-19 may not respond to a second dose of an mRNA vaccine and, hence, may not need it. In essence, the prior bout of COVID-19 may have provided sufficient immune stimulation such that the first dose of vaccine elicited a maximal or near maximal response.

In conclusion, the present study indicates that humoral responses to an mRNA vaccine are time dependent and differ in individuals with prior SARS-CoV-2 infection and infection-naive individuals. Individuals with prior, generally moderate-to-severe COVID-19 disease achieve a rapid, maximal or near maximal level of humoral immunity after a single dose of a COVID-19 mRNA vaccine. In fact, the humoral immune response to the second dose is greatly attenuated if not absent in individuals with prior COVID-19 disease.

The possibility that a single dose of vaccine in individuals with prior COVID-19 disease is as efficacious as the 2-dose regimen in achieving a protective immune response has profound public health implications. It affords an opportunity to conserve millions of doses that could be used to help address the critical worldwide shortage of vaccine. However, recent observations indicate that neutralizing activity against SARS-CoV-2 variants of concern is reduced in individuals with prior infection but is augmented by repeated vaccination (28). Accordingly, decisions regarding single versus double-dose vaccination in individuals with prior SARS-CoV-2 infection require controlled trials comparing protection achieved against infection with variants of concern with one versus 2 doses of vaccine.

## Methods

### Study design.

Individuals recruited (*n* = 61) into this vaccine study represented a subset of a larger cohort of healthcare workers (i.e., physicians, nurses, respiratory therapists, or other ancillary health care personnel; *n* = 281) participating in a surveillance study of SARS-CoV-2 spike RBD IgG seropositivity in our multihospital Health System. Individuals in the vaccine substudy agreed to have blood samples drawn at 2-week intervals for 56 days following the initial dose of the BNT162b2 mRNA vaccine. Individuals completed a questionnaire detailing their history of SARS-CoV-2 infection/COVID-19 disease, job description, demographics, and comorbidities.

Individuals in the SARS-CoV-2 infection/COVID-19–positive group (*n* = 30) had a documented history of COVID-19 with a positive nasopharyngeal swab for virus RNA or were seropositive for IgG antibody against the spike RBD or nucleocapsid proteins. Individuals in the infection-naive group (control group; *n* = 31) did not have COVID-19 illness or viral RNA detected by PCR testing and were IgG seronegative for the spike RBD and the nucleocapsid proteins.

The BNT162b2 vaccine was given to all individuals as currently recommended, i.e., two 30 μg, 0.5 ml intramuscular injections given 3 weeks apart. Vaccine administration in both groups took place from December 16, 2020, until April 2, 2021. Blood samples were obtained at 14, 28, 42, and 56 days after first dose. Those sampling intervals were based on the BNT162b2 phase I trial, which demonstrated maximal immune responses by day 42 (5).

### Patient and public involvement statement.

All participants were health care workers at our institution. Patients and the general public were not involved in any aspect of the study. Participants helped with recruitment by providing word of mouth recommendations to their coworkers and colleagues but were not involved in the design, conduct, reporting, or dissemination of our research.

Participants were provided with a written report of their individual data at regular intervals and again at the completion of the 56-day study period. Questions regarding results were encouraged and most participants made use of that option. Anonymized group data was also presented formally at departmental meetings to inform the participants of the study results.

### SARS-CoV-2 antibody.

Serum SARS-CoV-2 spike RBD IgG antibodies were quantified by a 2-step immunoassay using the Beckman Coulter Access microparticle-based system, run on a high-throughput UniCel Dxl 800 device (Beckman Coulter). This assay uses antigen-coated paramagnetic particles, which when mixed with participant serum, create an antigen-antibody complex. Anti-human IgG acridinium-labeled conjugate is then added to create a chemiluminescent signal measured as relative light units (RLU). Serum samples were diluted 1:10 and 1:20 to ensure that signals remained in the linear range of the standard curve. The system has a 4 orders of magnitude dynamic range (30, 31).

A recent study by Bartsch et al. indicates that spike RBD IgG antibody concentration above a critical threshold correlates with other aspects of immune function, such as serum neutralization activity, opsonization activity, and T cell activation responses to SARS-CoV-2 virus antigen (32). As such, the concentration of spike RBD IgG antibody seems to reflect the overall activity of the immune system. Accordingly, we used a recombinant, human IgG1 monoclonal SARS-CoV-2 spike RBD antibody, clone CR3022, produced in Nicotiana benthamiana (BEI Resources) to convert RLU values to μg/mL protein, as previously described (32, 33).

SARS-CoV-2 nucleocapsid IgG antibody was also measured to detect possible superimposed SARS-CoV-2 infection during the 56 days after vaccination. Nucleocapsid IgG antibody was measured using the Abbott Alinity system, which is also a 2-step microparticle chemiluminescent immunoassay.

### Neutralization assay.

Serum neutralization assays were performed as previously described using luciferase-expressing lentiviral particles pseudotyped for the SARS-CoV-2 spike protein and HEK293T cells overexpressing the ACE2 receptor (see Supplemental Methods and Supplemental Figures 3 and 4 for further details) (34, 35). The spike protein was from SARS-CoV-2 strain Wuhan-Hu-1 as previously described by Crawford et al. (34). Neutralizing activity was expressed as the serum dilution which produced IC_50_ of pseudoparticle entry. IC_50_ was calculated using sigmoidal 4 factor polynomial, nonlinear regression in GraphPad Prism (version 9).

### Vaccine reactogenicity.

The duration and severity of local and systemic reactions to the vaccine that occurred within 7 days of each injection were assessed using a standard questionnaire administered at the time of each blood draw. The severity of solicited local (pain/tenderness) and systemic reactions (fever, malaise, headache) were scored from 0 (none) to 10 (most severe), with scores of ≥6 classified as severe.

### Statistics.

Continuous measures within groups were expressed as mean ± 1 SEM. Comparison of categorical variables between groups was performed by Fisher’s exact test and within groups by McNemar’s test for paired comparisons. Comparison of changes in antibody levels over time or between the COVID-19 and control groups was assessed by linear mixed-effects models for repeated measures. Statistical significance of differences was accepted at the *P* < 0.05 level.

### Study approval.

The study was approved by the Temple University Institutional Review Board (IRB 27321). All individuals provided written informed consent.

## Author contributions

SGK and ASB designed and conceptualized the study. MOA, PSP, CR, JAH, and ASB collected the data. SGK, ASB, MOA, CR, and PSP analyzed the data. MRR, SGF, HZ, and NTG reviewed the analysis. SGK and MOA wrote the first draft of the manuscript with input from ASB, SGF, HZ, MRR, and NTG. All authors contributed to the final draft.

## Supplementary Material

Supplemental data

ICMJE disclosure forms

## Figures and Tables

**Table 1:**
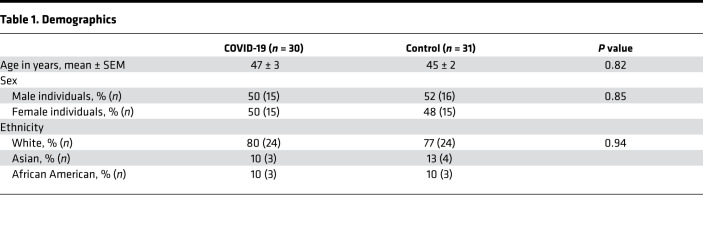
Demographics

**Table 2:**
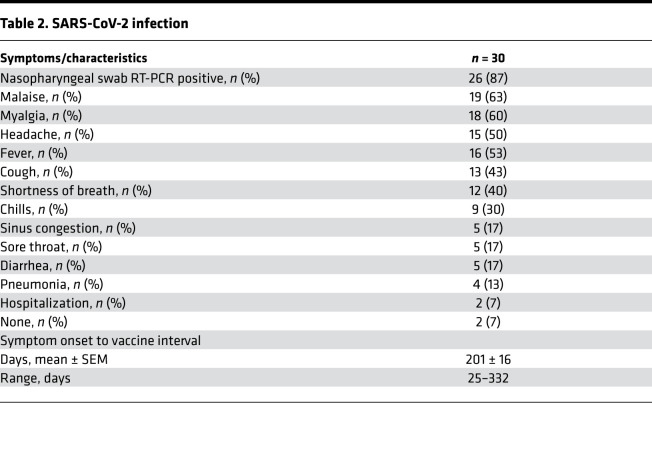
SARS-CoV-2 infection

**Table 3:**
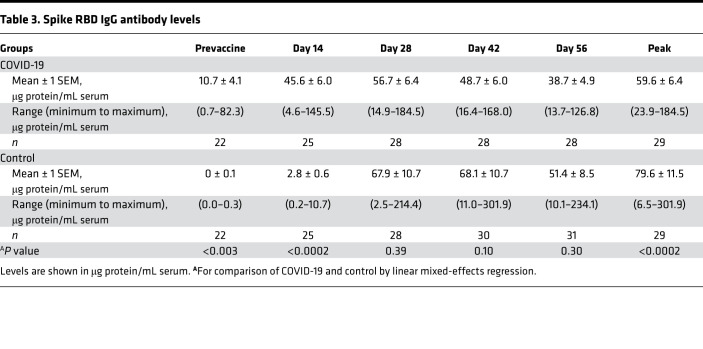
Spike RBD IgG antibody levels

## Version 1 01/12/2022

In-Press Preview

## Version 2 02/22/2022

Electronic publication
