# Supplementary material for: SARS-CoV-2 BNT162b2 vaccine–induced humoral response and reactogenicity in individuals with prior COVID-19 disease
Source: JCI Insight. 2022 Feb 22;7(4):e155889. doi: 10.1172/jci.insight.155889 (PMC8876462; doi:10.1172/jci.insight.155889)
Supplement: Supplemental data [file jciinsight-7-155889-s123.pdf]

SUPPLEMENTAL MATERIAL

METHODS - NEUTRALIZATION ASSAY

Serum neutralization assays were performed as previously described by Crawford et al using luciferase-expressing lentiviral particles pseudotyped for the SARS-CoV-2 spike protein (SARS-Related Coronavirus 2, Wuhan-Hu-1 Spike-Pseudotyped Lentiviral Kit, BEI Resources, NR-52948) and HEK-293T cells over-expressing the ACE2 receptor (HEK-293T-hACE2, BEI Resources, NR-52511) (34).

Pseudovirus preparation – Pseudotyped lentivirus was generated in HEK-293T cells transfected simultaneously with the following: helper plasmids encoding for Gag and Pol, Tat1b, and Rev1b; lentiviral backbone plasmid encoding for Luc2 and ZsGreen; and plasmid encoding the Wuhan-Hu-1 strain of SARS-CoV-2 Spike glycoprotein (Lentiviral Kit, BEI Resources, NR-52948). After 72 hrs, the culture supernatant containing pseudovirus was harvested, syringe-filtered (0.45 µm), and stored at -80° C.

Neutralization assay - HEK-293T-hACE2 cells (80,000 cells/well; BEI Resources, NR-52511) were cultured in white-walled microplates for 24 hr. Pseudovirus entry was assessed from luciferase activity using a GloMax Discover luminometer (Promega Corp., GM3000) and Bright-Glo reagent (Promega, E2610). Initially, to determine optimum conditions for the assay, the signal generated by various pseudovirus dilutions was assessed in the absence of serum **(Fig. S3).** Negative controls i.e., supernatants from HEK-293T cells transfected with carrier DNA only (Promega, E4881) (data not shown), or with a lentiviral plasmid lacking the Spike glycoprotein, yielded RLU values less than 0.2% of the undiluted pseudovirus (**Fig. S3**). For the neutralization assay itself, a 10x-dilution of pseudovirus stock (plus 5 µg/mL polybrene, EMD Millipore Corp., TR-2003-G) which yielded an RLU of 25,000 was used. Seven-point serial serum dilutions were incubated with pseudovirus for 1hr at 37°C in a separate sterile plate.

The serum-pseudovirus mixtures were then incubated with HEK-293T- hACE2 cells for 48 hr at 37°C, followed by lysis with Bright-Glo reagent. A potent neutralizing Spike RBD IgG antibody (BEI Resources, NR 53795) was used as a positive control in each run (**Fig. S4**).

Experimental Design and Data Analysis - Neutralization assays were performed pre-vaccination and at days 14 and 42 post 1st injection of vaccine. Individual serum samples (50 µL / subject) were pooled with 7 subjects per pool in both COVID and control groups. On days 14 and 42, data points for both groups are the mean ± SE of 3 pools of the same 21 COVID and 21 control subjects. In contrast, the pre-vaccination timepoint was a single pool for each group.

Maximal pseudovirus uptake by HEK cells in the absence of serum was taken as 100% of control. IC_{50} i.e., 50% inhibition of pseudovirus entry, was calculated using sigmoidal 4 factor polynomial, non-linear regression (GraphPad Prism version 9).

ACKNOWLEDGEMENTS

The following reagents were obtained through BEI Resources, NIAID, NIH: Monoclonal Anti-SARS Coronavirus Recombinant Human IgG1, Clone CR3022 (produced in Nicotiana benthamiana), NR-52392; SARS-Related Coronavirus 2, Wuhan-Hu-1 Spike-pseudotyped Lentiviral Kit, NR-52948; Human Embryonic Kidney Cells (HEK-293T) Expressing Human Angiotensin-Converting Enzyme 2, HEK-293T-hACE2 Cell Line, NR-52511; Monoclonal Anti-SARS-Related Coronavirus 2 Spike Glycoprotein RBD-mFc Fusion Protein (produced in vitro), NR-53795.

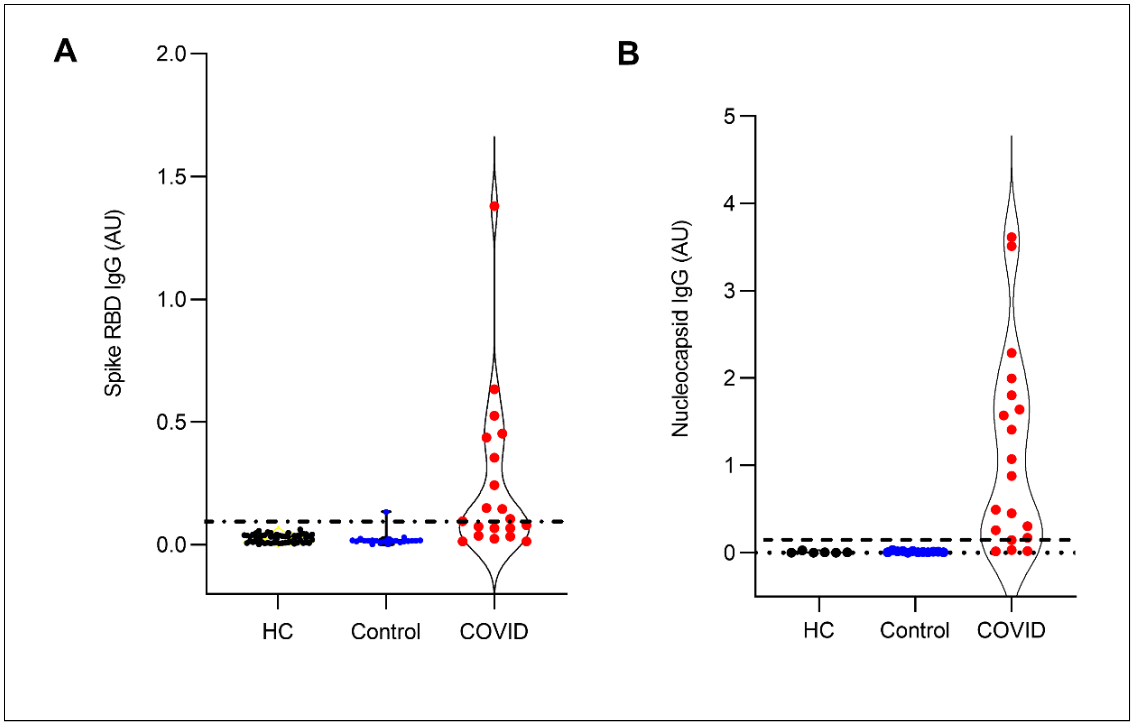

Figure S1. Violin plots showing pre-vaccination serum Spike RBD IgG antibody (panel A) and nucleocapsid IgG antibody (panel B) in individual COVID (●, n=22**) and control (NC●,** n=22**) subjects.** Spike RBD IgG (p<0.01) and nucleocapsid IgG (p<0.003) antibody levels were significantly higher in the COVID compared to control groups prior to vaccination. Historical control samples (**HC-●**; n=57) were archived prior to the COVID-19 pandemic i.e., 2008. Horizontal dashed line demarcates 4 standard deviations from the mean of the historical controls.

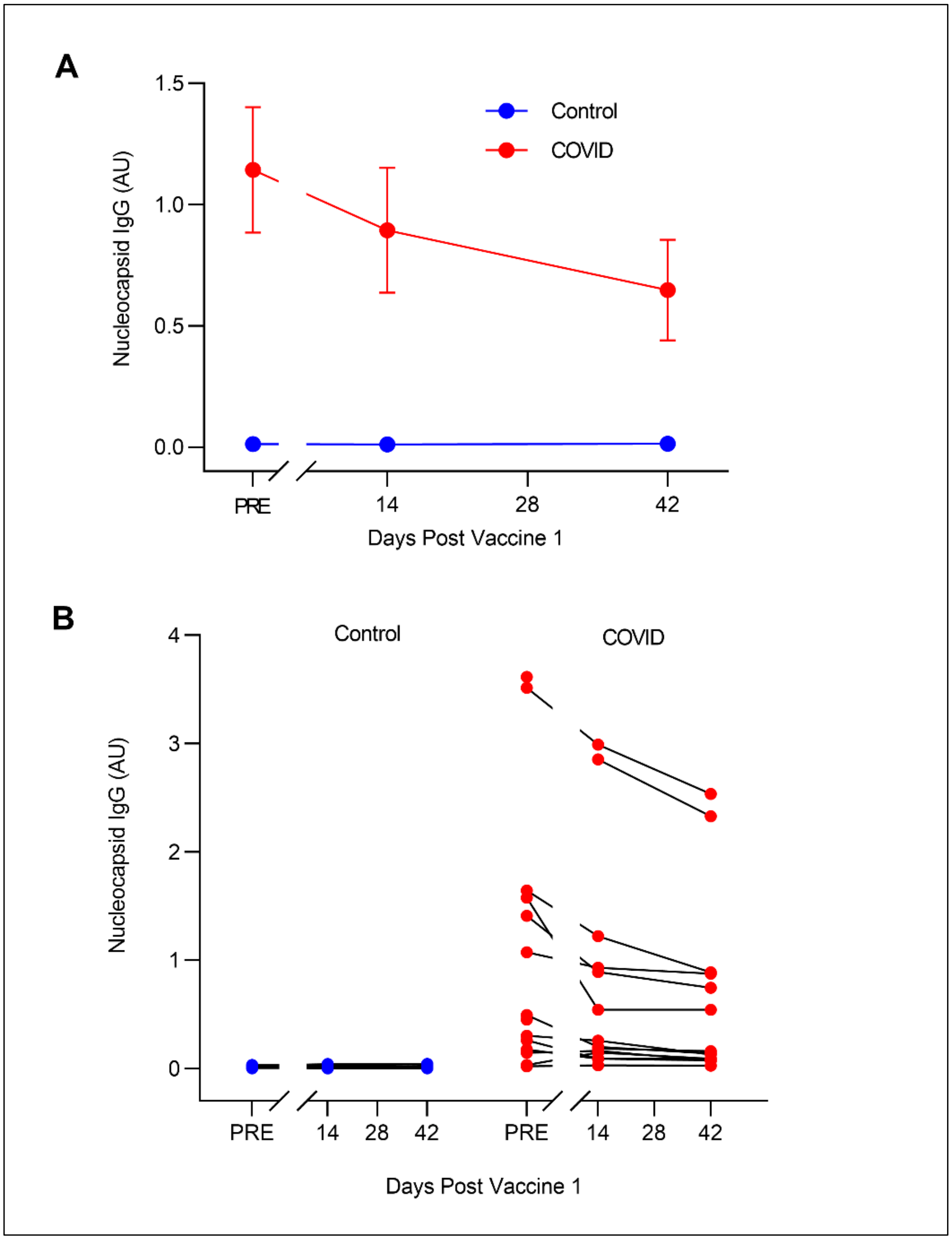

Figure S2. Nucleocapsid IgG antibody in COVID (●) and control (●) groups following the Pfizer BNT162b2 vaccine. Panel A: Shows group mean ± 1 SE responses. Sample size for COVID group was: day 14 (n=16), day 42 (n=18). Sample size for control group was: day 14 (n=16), day 42 (n=17). **Panel B:** Individual COVID and control subjects. Note that nucleocapsid IgG antibody is undetectable in the control group and falls over time post vaccination in the COVID group.

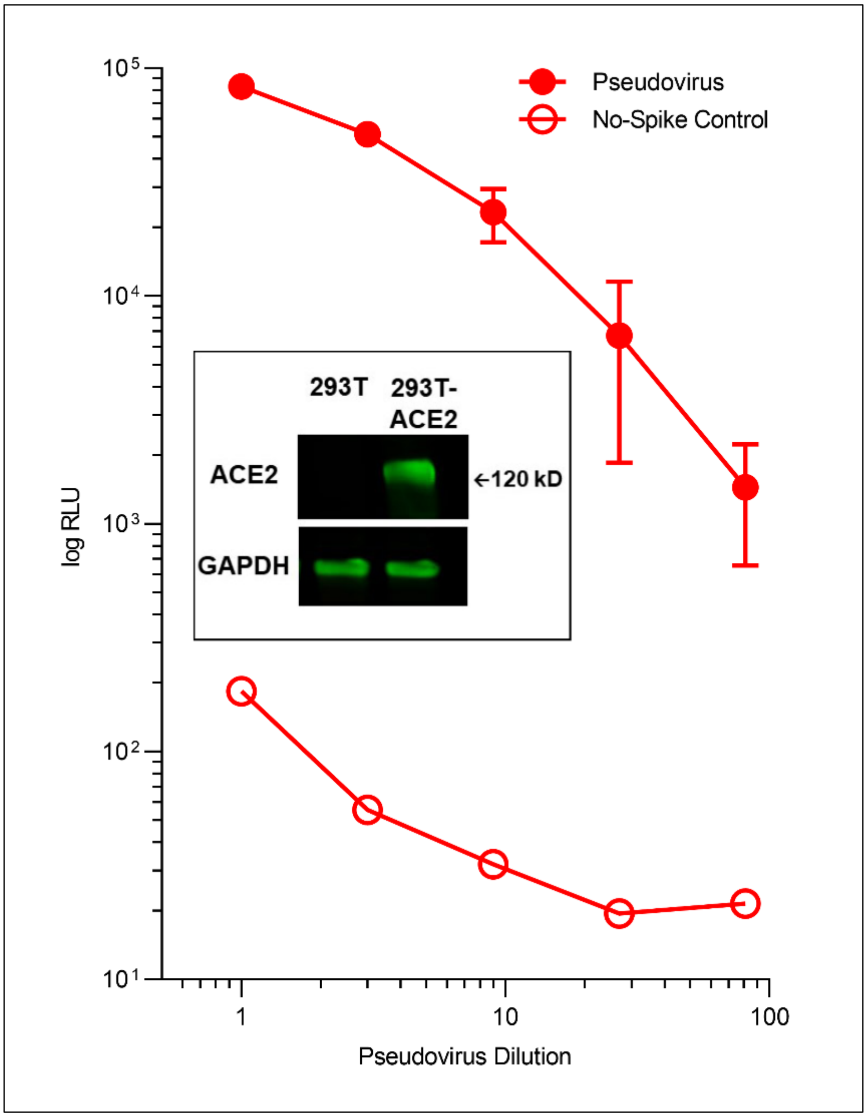

Figure S3. Uptake of pseudotyped SARS-CoV-2 lentivirus into HEK293T-ACE2 cells. Intact pseudotyped lentivirus (●) uptake shown as luciferase activity on the Y axis (relative light units - RLU) was assessed in the absence of serum. Pseudovirus dilutions are shown on the X axis. Lentivirus lacking SARS-CoV-2 Spike protein (○) was used as an inactive, negative control. Data are Group mean ± 1 SE from multiple wells (1 experiment representative of 3). Note that intact pseudovirus diluted 10X from stock generated a signal 500-fold greater than that produced by the inactive pseudovirus. Inset - Western blot showing marked increase in ACE2 receptor expression in 293T-ACE2 vs native 293T cells.

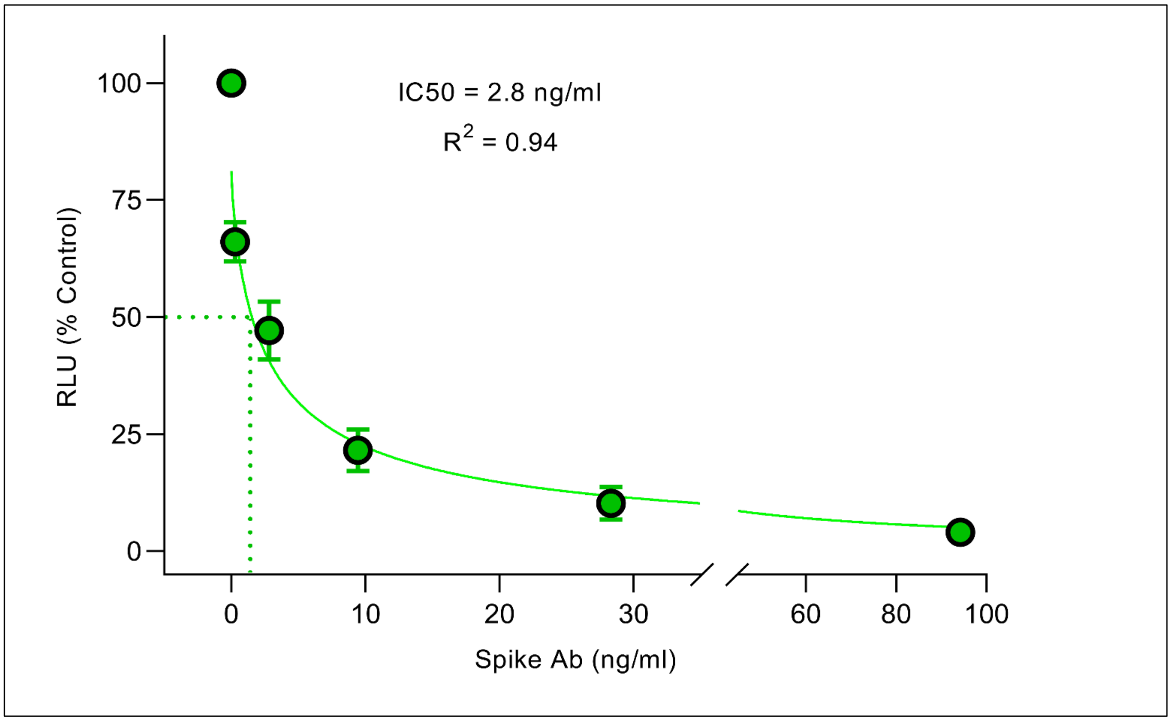

Figure S4. Effect of neutralizing Spike IgG antibody on pseudotyped SARS-CoV-2 lentivirus entry into HEK293T-ACE2 cells. Note that positive control Spike RBD neutralizing antibody (BEI Resources, NR-53795) strongly inhibited pseudovirus uptake (IC_{50}=2.8 ng/mL). Data are mean ± 1 SE of 4 experiments.
